# Trends and Association of Environmental Exposure and Climate Change with Non-Communicable Diseases in Latin America

**DOI:** 10.3390/healthcare13141653

**Published:** 2025-07-09

**Authors:** Andrés Alvarado-Calvo, Yazlin Alvarado-Rodríguez, Kevin Cruz-Mora, Jeaustin Mora-Jiménez, Sebastián Arguedas-Chacón, Esteban Zavaleta-Monestel

**Affiliations:** 1 Quality and Environment Management, Clínica Bíblica, San José 1307-1000, Costa Rica; aalvarado@clinicabiblica.com; 2 Medical Education, Clínica Bíblica, San José 1307-1000, Costa Rica; yalvarador@clinicabiblica.com; 3 Pharmacy Department, University of Costa Rica, San José 1307-1000, Costa Rica; kevin.cruzmora@ucr.ac.cr; 4 Pharmacy Department, Clínica Bíblica, San José 1307-1000, Costa Rica; jmoraj@clinicabiblica.com; 5 Health Research Department, Clínica Bíblica, San José 1307-1000, Costa Rica; sarguedas@clinicabiblica.com

**Keywords:** climate change, non-communicable diseases, air pollution, disability-adjusted life years, Latin America

## Abstract

**Background/Objectives**: Climate change is a major factor exacerbating non-communicable diseases (NCDs) such as cardiovascular diseases, neoplasms, respiratory diseases, and diabetes, especially in vulnerable Latin American regions. This study analyzes the impact of environmental exposures related to climate change on the NCD burden in eight Latin American countries by quantifying the disability-adjusted life years (DALYs) attributable to these factors. Using Global Burden of Disease (GBD) data (1990–2021), we performed multiple linear regression to assess associations between DALYs and environmental risk factors—air pollution (particulate matter, nitrogen dioxide), radon, lead, and extreme temperatures—in Argentina, Brazil, Chile, Colombia, Costa Rica, Mexico, Peru, and Uruguay. The study included major NCDs, and the population was stratified by age and sex. **Results**: Ischemic heart disease was the leading cause of DALYs in most countries. Particulate matter pollution was the main environmental risk factor contributing to the NCD burden, mainly affecting cardiovascular and respiratory diseases. Mexico showed the highest DALYs from particulate and ozone pollution; temperature and lead exposure also contributed in some countries. Nitrogen dioxide was the primary risk factor for asthma. Statistically significant relationships between environmental factors and DALYs were confirmed. **Conclusions**: Climate change-related exposures significantly increase the burden of NCDs in Latin America. Targeted interventions in industry, transportation, and energy, along with sustainable urban policies, are essential to mitigate health impacts and reduce disparities. Integrating environmental health into public policies can improve health outcomes amid ongoing climate challenges.

## 1. Introduction

Climate change significantly influences non-communicable diseases (NCDs) through multiple mechanisms. Factors such as the rise in extreme temperatures and air pollution exacerbate the burden of NCDs, particularly affecting the most vulnerable populations and regions [1]. Environmental exposure has a considerable impact on the global burden of non-communicable diseases, as exposure to fine particulate matter—particularly that associated with air pollution and climate change—has been shown to play a key role in their exacerbation [2].

There are four main types of non-communicable diseases, which include cardiovascular diseases (CVDs), neoplasms, chronic respiratory diseases, and diabetes [2]. The rise in these NCDs is attributable to critical influencing factors such as air pollution, temperature changes, and extreme conditions like floods or droughts. These conditions negatively impact food security, which in turn affects diet and increases the risk of developing these diseases [3].

In urban settings of low- and middle-income countries, climate change intensifies the challenges of managing NCDs [4]. Latin America faces a dual challenge due to the intersection of climate change and the rise of NCDs, which represent an increasing burden on healthcare systems [5]. With climate change accelerating extreme weather events and altering environmental conditions, the region is increasingly affected by the rise in respiratory, cardiovascular, and metabolic diseases. These adverse effects are further exacerbated by socioeconomic factors and increasing urbanization [6,7].

Although region-wide data for Latin America are limited, available estimates indicate that non-communicable diseases and mental health disorders already account for approximately 4% of the gross domestic product (GDP) in South America between 2020 and 2025, representing an economic loss of nearly USD 7.3 trillion [8].

This impact is not exclusive to the region. In high-income economies such as the United States, NCDs are projected to cause a loss of 94.9 trillion dollars between 2015 and 2050, with cardiovascular diseases and mental disorders being the main contributors, followed by cancer, diabetes, and chronic respiratory diseases [9]. Similarly, in China, air pollution is estimated to result in economic losses of USD 1.137 trillion between 1990 and 2030, with cardiovascular diseases once again leading the burden, followed by chronic respiratory diseases, diabetes, and cancer [10].

These figures highlight that the economic burden of NCDs is substantial and global, reinforcing the urgency of adopting mitigation strategies that integrate public health, environmental policies, and sustainable economic development. Unlike previous reports based on aggregated GBD data, this study offers a novel contribution by quantifying and comparing the specific burden of environmental risk factors—including air pollution, temperature extremes, radon, and lead—on NCDs across eight individual Latin American countries. By applying multiple linear regression models to estimate the association between these exposures and disability-adjusted life years (DALYs), stratified by sex and age group, our work reveals distinct national patterns and risk profiles that are not evident in broader regional analyses. This fine-grained approach allows for a deeper understanding of country-specific vulnerabilities and can inform targeted public health and environmental policy interventions.

## 2. Materials and Methods

### 2.1. Data Source

For this analysis, data from the Global Burden of Disease (GBD) study were used. These data provide standardized estimates of health indicators for multiple diseases and injuries across 204 countries and territories [11]. The GBD study enables a detailed assessment and measurement of health loss and trends in NCDs. This analysis included the six main non-communicable conditions: ischemic heart disease, ischemic stroke, neoplasms, asthma, chronic obstructive pulmonary disease (COPD), and diabetes.

### 2.2. Study Population and Time Frame

This study focuses on eight Latin American countries: Argentina, Brazil, Chile, Colombia, Costa Rica, Mexico, Peru, and Uruguay. These nations were selected based on the availability of comparable data in the GBD database and for representing geographic, economic, and climatic diversity within the region. The countries were chosen according to their level of industrialization and vulnerability as determined by the ND-GAIN Index [12].

Our study was limited to an analysis covering the period from 1990 to 2021, using estimates for all ages and both sexes, with a focus on the age groups 15–49 years, 50–69 years, and 70 years and older. Environmental risk factors related to climate change were also incorporated, including air pollution (from fine particulate matter and nitrogen dioxide), exposure to radon and lead, as well as extreme temperatures. This population stratification allows for a precise analysis of the inequalities in disease burden attributable to climate change.

### 2.3. Health Metrics Analyzed

DALY is an indicator that reflects the total loss of healthy life in a population, combining premature mortality and years lived with disability due to diseases. This indicator is calculated using the following formula:

DALY = YLL + YLD(1)

where YLL (Years of Life Lost) measures the years lost due to premature mortality, and YLD (Years Lived with Disability) assesses the burden of diseases that do not necessarily cause death but do impair quality of life. Both indicators are expressed per 100,000 inhabitants and are age-standardized [13,14,15].

DALY provides a comprehensive measure of the loss of healthy life, taking into account both mortality and morbidity. This indicator is useful for evaluating the overall impact of diseases on a population, as it includes not only premature deaths but also the effects of chronic and disabling conditions that impair quality of life, such as diabetes or respiratory diseases. Thus, DALY offers a complete view of the disease burden, encompassing both mortality and the associated disability [13,14,15].

Accordingly, the values of YLL, YLD, and DALY used in this study were taken directly from the open-access GBD 2021 repository. Within this framework, YLLs are calculated by multiplying the number of deaths by cause, sex, and age group by the standard life expectancy table, while YLDs are obtained by multiplying the prevalence of each sequela by its disability weight—parameters estimated through the Bayesian tool DisMod-MR 2.1 and health valuation surveys. Since GBD 2010, these metrics have been published without age-weighting or discounting and are age-standardized using the GBD’s own global reference population. Therefore, these indicators were not recalculated; instead, the point estimates and their 95% uncertainty intervals (95% UI) for the eight selected countries were downloaded and directly used as dependent variables in the multiple linear regression model [16].

### 2.4. Statistical Analysis

In the analysis conducted, a multiple linear regression model was used to calculate the DALY values in relation to various variables such as disease causes, risk factors, age, sex, and geographic location. This approach allows for the assessment of the impact of independent variables on DALYs—that is, the number of years lost due to diseases and other conditions. The analysis included two separate models: one adjusted for each country and another that considered the variables independently. The results showed that all examined variables had a statistically significant relationship with DALYs, supporting the validity of the model used.

## 3. Results

Using data from GBD 2021, this analysis examines NCDs in eight Latin American countries during the period from 1990 to 2021. The countries selected for the analysis were Argentina, Brazil, Chile, Colombia, Costa Rica, Mexico, Peru, and Uruguay, based on the availability of complete and comparable data. The key indicator extracted and analyzed was DALY, in order to provide a comprehensive understanding of the NCD burden, reflecting both premature mortality and disability associated with these diseases, expressed per 100,000 inhabitants. The following are the results obtained from these data, with a focus on trends and regional variations.

**Figure 1:**
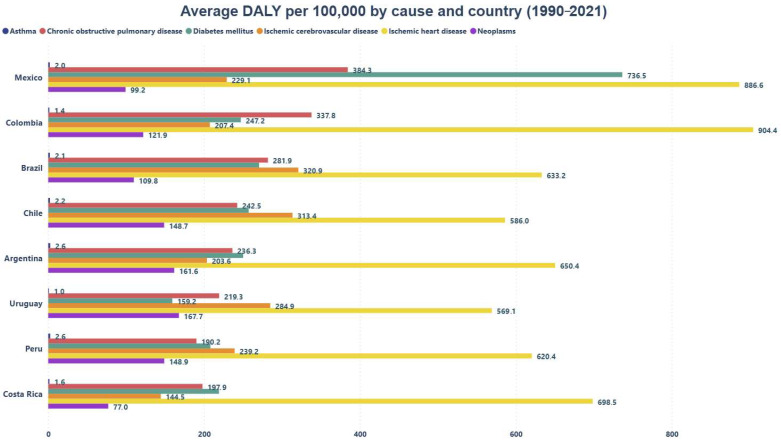
Average DALYs per 100,000 by Cause and Country.

Figure 1 shows a common trend across all countries, where ischemic heart disease is the condition with the greatest impact on DALYs in most of the selected samples. On the other hand, ischemic stroke also has a notable impact on cardiovascular health, being especially relevant in countries such as Brazil, Chile, Peru, and Uruguay, where it ranks as the second leading condition affecting DALYs.

Additionally, it is noteworthy that Mexico is the only country where diabetes mellitus ranks second in terms of DALY burden. In Colombia, COPD is the condition with the highest impact on DALYs. Finally, asthma consistently shows the lowest DALY burden across all the countries analyzed.

**Figure 2:**
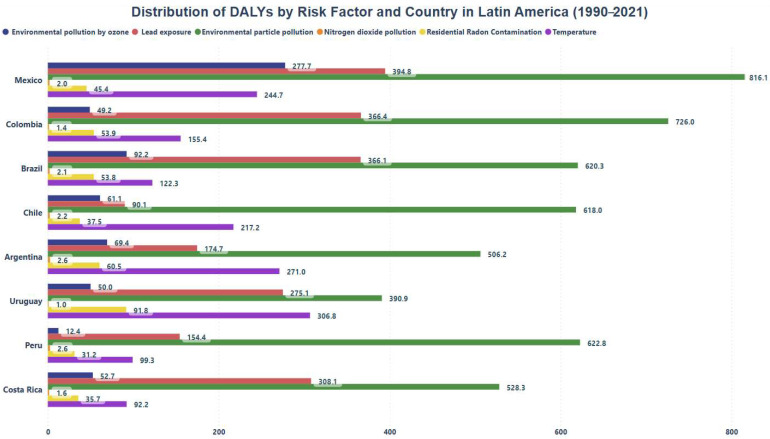
Distribution of DALYs by Risk Factor and Country in Latin America.

Figure 2 shows that ambient particulate matter pollution has the greatest impact on DALYs across all analyzed countries, with Mexico being the most affected, registering 816.1 (CI 95% 746.66–885.47) DALYs. Additionally, Mexico shows a significant impact from ozone pollution with 277.7 (CI 95% 226.5–328.9) DALYs, while Peru has the lowest impact on this indicator, with only 12.4 (CI 95% 9.8–15.0) DALYs.

On the other hand, countries such as Mexico, Colombia, Brazil, Peru, and Costa Rica exhibit notable DALYs values associated with lead exposure, registering 394.8 (CI 95% 343.3–446.2), 366.4 (CI 95% 314.5–418.4), 366.1 (CI 95% 326.5–405.6), 154.4 (CI 95% 135.6–173.2), and 308.1 (CI 95% 265.0–351.2), respectively. Although these values are lower than those linked to particulate matter pollution, they still represent a considerable burden. Furthermore, in countries like Argentina, Uruguay, and Chile, temperature is the second most relevant risk factor for DALYs, significantly contributing to the loss of healthy life years in certain contexts across Latin America.

**Figure 3:**
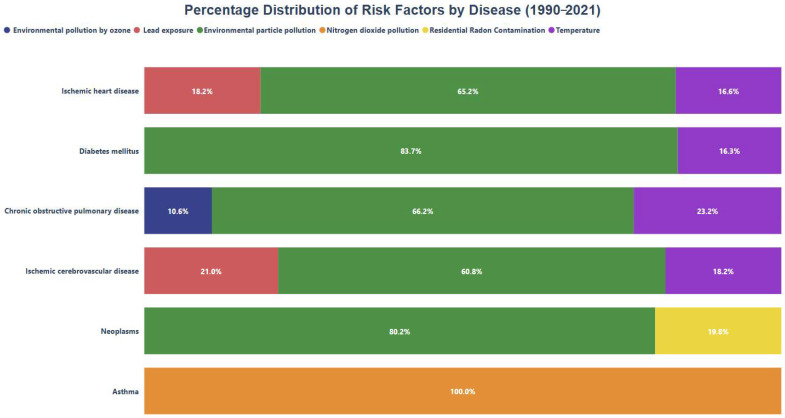
Percentage distribution of risk factors by condition.

Figure 3 shows that particulate matter pollution is the leading environmental risk factor associated with the development of conditions such as COPD, diabetes mellitus, neoplasms, ischemic heart disease, and ischemic stroke. This suggests that exposure to particulate matter plays a significant role in the onset of these diseases, emphasizing the importance of implementing preventive strategies to reduce such exposure and mitigate the overall burden of non-communicable diseases.

Regarding neoplasms, a significant portion of the burden is associated with residential radon exposure. On the other hand, although lead exposure is not the primary risk factor for any specific disease, it still holds considerable relevance in increasing disease burden, particularly in ischemic heart disease and ischemic stroke. Additionally, ozone (O_3_) pollution accounts for 10.60% of the COPD burden, while nitrogen dioxide (NO_2_) pollution constitutes 100% of the attributable risk factor for asthma, standing out as the sole relevant risk factor for this condition.

## 4. Discussion

### 4.1. Impact of Particulate Pollution

The analyzed data show that particulate matter pollution is the main risk factor for the various diseases studied. In general, particulate matter (PM) is classified by aerodynamic diameter into three categories: PM10, with a diameter greater than 10 μm, which deposits on mucous membranes and upper airways; PM2.5, fine particles approximately 2.5 μm in size, which easily penetrate the pulmonary alveoli; and PM0.1, ultrafine particles smaller than 0.1 μm, which are capable of crossing pulmonary membranes, being absorbed by cells and transported through the bloodstream—thus exposing all cells in the body to potential damage [17].

At the global level, particulate matter pollution ranks fifth among risk factors for mortality. Within the fine fraction, microplastics (MPs, <5 mm) and especially nanoplastics (NPs, <1 μm) have emerged as new contaminants. Urban studies have detected MPs/NPs in all PM2.5 samples, accounting for up to 12% of their mass and showing a strong correlation between PM2.5 concentration and atmospheric plastic load [18].

Inhalation is the primary route of entry for these plastics into the human body. Their submicrometric size allows them to cross respiratory barriers, translocate into the bloodstream, and bioaccumulate in various tissues. In addition to directly increasing the particulate matter burden, their high surface area enables them to adsorb and transport heavy metals and toxic organic compounds, thereby amplifying oxidative stress [19].

Another key factor associated with PM pollution is epigenetics. Particulate matter induces epigenetic modifications primarily through DNA methylation, histone remodeling, and changes in microRNAs, which alter gene expression in target tissues and serve as a molecular axis in the development of NCDs. Chronic exposure to PM generates oxidative stress and sustained inflammation; both signals trigger the epigenetic reprogramming of genes involved in inflammation, immunity, and tissue repair [20].

**Figure 4:**
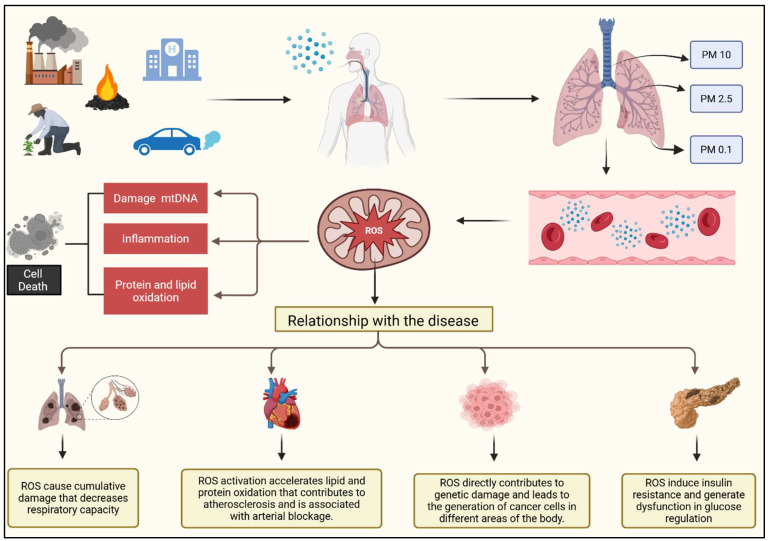
Biological mechanisms of ambient particulate matter pollution and its relationship with non-communicable diseases. Exposure to particulate matter (PM) generates reactive oxygen species (ROS), which cause mitochondrial DNA damage, trigger inflammation, and promote protein and lipid oxidation. These processes contribute to cumulative respiratory damage, cardiovascular diseases through oxidative stress and atherosclerosis, genetic damage linked to cancer development, and metabolic dysfunction such as insulin resistance and type 2 diabetes [22,23,24,25,26,27,28,29,30].

Figure 4 illustrates how fine particulate matter pollution triggers processes that lead to the release of reactive oxygen species (ROS). These molecules are primarily generated in the mitochondrial matrix in the form of superoxide (O_2_) and hydrogen peroxide (H_2_O_2_), but environmental factors disrupt this process, leading to increased ROS production [21].

The accumulation of ROS in the mitochondria causes structural changes, such as altered mitochondrial membrane permeability, and damages mitochondrial DNA, increasing mutations. It also leads to the oxidation of proteins and lipids. This, in turn, compromises the function of the mitochondrial respiratory chain, interferes with ATP production, and ultimately results in cell death [21].

### 4.2. Relationship Between Particulate Pollution and Non-Communicable Diseases

#### 4.2.1. Cardiovascular Diseases

The disease with the greatest impact on DALYs in this analysis was ischemic heart disease. This finding aligns with trends reported in the literature, confirming it as the leading contributor to the disease burden reflected in DALYs. Globally, ischemic heart disease represents the highest burden among all cardiovascular diseases, with 2275.9 DALYs per 100,000 population [22].

Its potential impact on DALYs may be attributed to the fact that PM harms the cardiovascular system through several key mechanisms. First, PM-induced oxidative stress and inflammation generate ROS and inflammatory cytokines that damage the vascular endothelium and promote atherosclerosis. Additionally, the resulting endothelial dysfunction can cause vasoconstriction, impairing vasomotor function and increasing the risk of hypertension and acute cardiovascular events. An imbalance in the autonomic nervous system is also observed, favoring cardiac arrhythmias. Ultrafine particles can translocate into the circulation, contributing to the progression of cardiovascular diseases, while elevated levels of endothelin-1—a potent vasoconstrictor—raise blood pressure and impair cardiac function [23,24,25].

#### 4.2.2. Diabetes Mellitus

Regarding type 2 diabetes mellitus (T2DM), it was found that countries such as Mexico had a greater impact on DALYs compared to the other countries analyzed. However, studies like that of Ilic & Ilic (2024) [26], based on GBD 2019 data, show that the DALY burden from T2DM in Latin America continues to follow an upward trend. In 2019, approximately 8.55 million DALYs were recorded (1169 per 100,000 population), with the highest age-standardized rate observed in Central America (1746 per 100,000).

Studies in both humans and animals have shown that air pollution negatively affects hepatic metabolism, particularly glucose homeostasis [27]. In an experiment with mice exposed to PM2.5 for 16 weeks, a decrease in glycolysis and Krebs cycle intermediates was observed, indicating a reduced capacity of this metabolic pathway—a characteristic finding in diabetes that is associated with insulin resistance [28].

#### 4.2.3. Chronic Obstructive Pulmonary Disease (COPD)

In the case of COPD, it was found that in 2019, it accounted for 942 DALYs per 100,000 inhabitants in Southern Latin America and 910 DALYs per 100,000 in Latin America as a whole. Combined, these two subregions represent approximately 3.2 million DALYs, or about 4% of the 74.4 million DALYs estimated globally for COPD that year. These trends are consistent with the findings of this study, as COPD was one of the leading contributors to DALYs in most countries, especially in countries like Colombia [27].

Traditionally, this disease has been associated with tobacco use; however, a significant proportion of individuals who develop COPD have never smoked. This has led to the investigation of other risk factors, such as PM exposure. Robust epidemiological studies have demonstrated a clear relationship between PM exposure and the development of COPD, highlighting key features such as chronic inflammation, oxidative stress, and airway remodeling. This oxidative stress, mediated by ROS, plays a fundamental role in exacerbating these processes, contributing to the progression of COPD in individuals exposed to particulate matter [29].

#### 4.2.4. Neoplasms

Exposure to fine particulate matter (PM), as well as its components such as metals, pollen, endotoxins, and polycyclic aromatic hydrocarbons (PAHs), activates cellular pathways involved in DNA repair. The ROS generated by these particles induces DNA damage, particularly in the form of oxidation, which can lead to the formation of 8-hydroxy-2′-deoxyguanosine and double-strand breaks. In addition, PAHs can form DNA adducts. This damage is associated with disruptions in DNA repair pathways, including base excision repair (BER), nucleotide excision repair (NER), and homologous recombination (HR). Although there is strong evidence that PM exposure causes DNA damage and alters these repair mechanisms, the efficiency of these processes and their biological consequences are still under investigation [30].

#### 4.2.5. Asthma and Its Relationship with Nitrogen Dioxide (NO_2_)

Studies based on GBD 2019 indicate that asthma continues to represent a substantial health burden in Latin America, with marked heterogeneity across countries, age groups, and sexes. Between 1990 and 2019, the region accumulated approximately 41.7 million DALYs attributable to asthma, with the highest rates concentrated among children under five years of age and adult women. These figures underscore the need for targeted prevention and management strategies that take into account the demographic and geographic variability of the disease [31].

Exposure to NO_2_ is closely linked to the exacerbation of allergic asthma through several mechanisms, as pollutants like NO_2_ act as environmental adjuvants, increasing epithelial mucosal permeability to external factors, thereby facilitating inflammation and differentiation toward a Th2 phenotype. This process promotes T-cell proliferation, cytokine secretion (such as IL-4, IL-5, and IL-13), mucus production, and airway remodeling—all hallmark features of allergic asthma. NO_2_ exposure also affects the immune balance between Th1 and Th2 cells. Th1 cells, which secrete IFN-γ, play a crucial role in inhibiting IgE production, thus reducing the allergic response. In turn, IFN-γ from Th1 cells can suppress IL-4 production in Th2 cells, limiting their activation and differentiation toward a pro-inflammatory phenotype. However, NO_2_ exposure does not directly disrupt this balance but rather worsens it, promoting the exacerbation of asthmatic inflammation and increasing disease severity [32].

### 4.3. Rising Temperatures Due to Climate Change and Their Impact on Non-Communicable Diseases

Climate change poses a major threat to public health worldwide in the coming decades. It is confirmed that without substantial efforts to limit greenhouse gas emissions, global average temperatures are projected to rise between 2.1 °C and 5.7 °C by the year 2100. This increase is expected to result in twice as many heatwaves in the coming decades compared to those observed at the end of the 20th century. These extreme temperatures can trigger heat stress and heatstroke—and in the most severe cases, even lead to death [33,34].

Rising temperatures have significant adverse effects on health. In cardiovascular diseases, elevated temperatures severely affect the cardiovascular system by accelerating blood circulation and heart rate, which in turn lowers blood pressure. The resulting cardiac output becomes insufficient to meet the body’s thermoregulatory demands, potentially leading to heat exhaustion, dehydration, and heatstroke—thereby increasing the risk of acute cardiovascular events [33,34].

In the case of respiratory diseases, high temperatures increase air pollutants and allergens, which worsen chronic respiratory conditions—especially in older adults. Heat exposure is also associated with a rise in hospitalizations due to respiratory illnesses. Regarding type 2 diabetes (T2DM), individuals with diabetes have reduced skin blood flow and a diminished sweating response compared to healthy individuals when exposed to heat, impairing their ability to dissipate heat effectively. This altered thermoregulation can negatively affect glycemic control, as it may lead to reduced insulin production and sensitivity, increasing the risk of hyperglycemia [33,34].

### 4.4. Integrated Public Policies: Energy, Urbanization, and Environmental Health

There are public policies promoted by international organizations such as the WHO, including the Global Action Plan for the Prevention and Control of Non-communicable Diseases 2013–2020, which has significantly contributed to mitigating environmental impact. This plan promotes measures such as the promotion of active transportation and urban planning with green infrastructure, both of which help reduce air pollution and greenhouse gas emissions [35].

Complementing this perspective, a study on renewable energy consumption in Latin America and the Caribbean between 1990 and 2016 revealed a nonlinear relationship between energy consumption, economic growth, urbanization, and mortality rates associated with air pollution. The study demonstrated that the use of renewable energy has a positive effect in reducing deaths caused by air pollution, due to its lower CO_2_ emissions [36].

In terms of policy implications, this same study recommends accelerating the energy transition toward renewable sources through public policies that aim not only to reduce pollution-related deaths but also to contribute to climate change mitigation. It also emphasizes that urbanization should not be restricted but rather qualitatively improved, steering it toward models of healthier and more sustainable cities [36].

### 4.5. Global Interventions to Mitigate Environmental Pollution and Their Importance in Latin America

#### 4.5.1. Industry-Targeted Interventions

The U.S. NOx Budget Trading Program set a cap on the total amount of nitrogen oxide (NOx) emissions allowed from industrial sectors, implementing a strict process for obtaining permits to emit these pollutants. This intervention led to a notable 40% reduction in emissions from power plants between 2000 and 2008, resulting in significant improvements in mortality rates and a decrease in PM10 levels. In contrast, China implemented the Two Control Zone Policy, which established mandatory industrial standards to control air pollution. However, evaluations of its impact on mortality did not show clear changes in outcomes, despite the efforts made in this regard [37,38].

#### 4.5.2. Residential Source-Targeted Interventions

Regarding residential source-targeted interventions, Dublin implemented a ban on the marketing, sale, and distribution of coal for heating, which resulted in a reduction in respiratory-related mortality [39]. However, no clear changes were observed in all-cause mortality or cardiovascular mortality. On the other hand, in California, an intermittent wood-burning ban was implemented in the San Joaquin Valley. This intervention was associated with a decrease in PM2.5 concentrations, demonstrating its effectiveness in improving air quality, although its impact on overall mortality remains unclear [40].

#### 4.5.3. Vehicle Emission-Targeted Interventions

In vehicle emission-targeted interventions, mandatory regulations were implemented in Tokyo and across Japan requiring older or more polluting diesel vehicles to be either replaced or equipped with advanced emission control systems. This measure was associated with improvements in all-cause mortality, as well as reductions in cardiovascular and respiratory mortality rates [41]. Additionally, in Beijing, China, a policy was implemented that restricts vehicle circulation based on license plate numbers (odd/even system). This strategy, which allows vehicles with even-numbered plates to circulate on certain days and those with odd-numbered plates on others, led to a significant reduction in PM10 concentrations. This improvement is attributed to the decreased number of vehicles on the road, which reduced emissions from vehicle engines [42].

#### 4.5.4. Multi-Source Targeted Interventions

The National Ambient Air Quality Standards (NAAQS) are a key component of the Clean Air Act, which regulates air quality and sets standards to protect human health and the environment from air pollution. The implementation of these measures did not show clear changes in all-cause mortality. However, there was a reduction in O_3_, PM2.5, and PM10 levels [43].

#### 4.5.5. Hospital-Targeted Interventions

In the healthcare sector, it has been identified that the overall health system contributes between 8% and 10% of all greenhouse gas emissions in the United States, and up to 25% of emissions in the public sector in the United Kingdom. Of this 8%, approximately 46% comes from direct healthcare activities and procurement, while 54% originates from indirect activities associated with the supply chain of healthcare-related goods and services [44,45].

Some of the interventions targeting the hospital sector to improve its environmental footprint include implementing strategies to reduce both direct and indirect emissions associated with healthcare delivery. These efforts not only enhance efficiency and service quality but also contribute to a reduction in adverse health effects. Initiatives such as the Healthier Hospitals Initiative (HHI) in the United States aim to improve sustainability through the adoption of renewable energy, energy efficiency, water conservation, waste reduction, and more environmentally responsible supply chain management. Furthermore, efforts to improve access to renewable energy and adopt environmentally friendly practices enable hospitals to reduce their impact on climate change while also improving public health by decreasing pollution-related deaths and illnesses [35].

#### 4.5.6. Relevance and Applicability of Interventions in Latin America

Interventions implemented in other countries to mitigate environmental pollution and improve air quality offer valuable lessons for Latin America. Strategies such as emission reductions in industrial sectors through programs like the U.S. NOx Budget Trading Program and vehicle control policies in Tokyo have proven effective in reducing pollutants and improving public health. Likewise, the ban on coal burning for heating in Dublin and vehicle circulation restrictions in Beijing are examples of measures that have led to improvements in air quality and respiratory mortality. Furthermore, the implementation of the National Ambient Air Quality Standards (NAAQS) in the U.S. and policies aimed at enhancing sustainability in hospitals can be adopted in Latin America to reduce the healthcare sector’s carbon footprint and improve public health conditions. Adopting similar approaches in the region could significantly contribute to mitigating the effects of climate change and improving air quality, thereby reducing the burden of NCDs associated with pollution.

## 5. Conclusions

This study addresses a critical intersection between climate change and non-communicable diseases (NCDs), offering a comprehensive view of how air pollutant emissions are exacerbating public health conditions in Latin America. The findings confirm that climate change—particularly in its manifestation as air pollution—is profoundly affecting the prevalence of cardiovascular, respiratory, and metabolic diseases in the region.

The analysis highlights how interventions in key sectors such as industry, transportation, and energy—focused on pollutant reduction and the transition to renewable energy—can mitigate this negative impact on health. Integrating these sustainable approaches into public policy is essential not only to improve air quality but also to reduce health disparities among the most vulnerable populations.

Moreover, the study underscores that environmental and sustainable urbanization policies are strategic tools that not only benefit the environment but also safeguard human health. This study sets an important precedent by demonstrating how proper climate change management can directly improve public health and reduce the burden of pollution-related diseases.

## 6. Limitations

This analysis presents several inherent limitations. First, its ecological design relies on country-level aggregates from the Global Burden of Disease (GBD) study, which prevents inference of causality at the individual level and may introduce the so-called ecological fallacy. Moreover, exposure metrics for particulate matter, NO_2_, radon, lead, and temperature are derived from national averages or models, and therefore do not capture subnational or micro-environmental variability; as a result, actual exposure levels may be under- or overestimated.

Second, the exposure–response functions and counterfactual levels used to attribute risk were drawn from the global literature. While widely accepted, they may not fully capture the specific pollutant mixtures, susceptibilities, and socioeconomic contexts of Latin American populations. Additionally, although adjustments were made for age, sex, and country, it was not possible to control for all potential confounding factors (e.g., smoking prevalence, diet, access to healthcare). The combination of modeled GBD inputs and limited covariate adjustment widened the uncertainty intervals, reducing the precision of some associations and highlighting the need for future studies using individual-level data, more granular exposure measurements, and more robust statistical methods.

## Data Availability

The data supporting the findings of this study are available from the corresponding author upon reasonable request. Due to privacy and ethical restrictions, the data are not publicly available. However, aggregated summary data are included within the manuscript.

## Abbreviations

The following abbreviations are used in this manuscript:

COPD	Chronic Obstructive Pulmonary Disease
CVD	Cardiovascular Disease
DALY	Disability-Adjusted Life Year
GBD	Global Burden of Disease
GDP	Gross Domestic Product
HHI	Healthier Hospitals Initiative
IL	Interleukin
IFN-γ	Interferon Gamma
NAAQS	National Ambient Air Quality Standards
NCDs	Non-Communicable Diseases
NO_2_	Nitrogen Dioxide
NOx	Nitrogen Oxides
O_3_	Ozone
PAHs	Polycyclic Aromatic Hydrocarbons
PM	Particulate Matter
PM2.5	Particulate Matter with a diameter ≤ 2.5 µm
PM10	Particulate Matter with a diameter ≤ 10 µm
ROS	Reactive Oxygen Species
Th1	T-helper Type 1 Cell
Th2	T-helper Type 2 Cell
YLD	Years Lived with Disability
YLL	Years of Life Lost

## References

[1] Münzel T., Hahad O., Sørensen M., Lelieveld J., Duerr G.D., Nieuwenhuijsen M., Daiber A. (2022). Environmental risk factors and cardiovascular diseases: A comprehensive expert review. Cardiovasc. Res..

[2] Siiba A., Kangmennaang J., Baatiema L., Luginaah I. (2024). The relationship between climate change, globalization and non-communicable diseases in Africa: A systematic review. PLoS ONE.

[3] Khraishah H., Alahmad B., Ostergard R.L., AlAshqar A., Albaghdadi M., Vellanki N., Chowdhury M.M., Al-Kindi S.G., Zanobetti A., Gasparrini A. (2022). Climate change and cardiovascular disease: Implications for global health. Nat. Rev. Cardiol..

[4] Rother H.A. (2020). Controlling and preventing climate-sensitive noncommunicable diseases in urban sub-Saharan Africa. Sci. Total Environ..

[5] Kephart J.L., Sánchez B.N., Moore J., Schinasi L.H., Bakhtsiyarava M., Ju Y., Gouveia N., Caiaffa W.T., Dronova I., Arunachalam S. (2022). City-level impact of extreme temperatures and mortality in Latin America. Nat. Med..

[6] Laborde A., Tomasina F., Bianchi F., Bruné M.-N., Buka I., Comba P., Cori L., Duffert C.M., Harari R., Iavarone I. (2015). Children’s health in Latin America: The influence of environmental exposures. Environ. Health Perspect..

[7] Asadi-Lari M., Ahmadi Teymourlouy A., Maleki M., Afshari M. (2021). Opportunities and challenges of global health diplomacy for prevention and control of noncommunicable diseases: A systematic review. BMC Health Serv. Res..

[8] Ferranna M., Cadarette D., Chen S., Ghazi P., Ross F., Zucker L., Bloom D.E., Dávila-Cervantes C.A. (2023). The macroeconomic burden of noncommunicable diseases and mental health conditions in South America. PLoS ONE.

[9] Chen S., Kuhn M., Prettner K., Bloom D.E. (2018). The macroeconomic burden of noncommunicable diseases in the United States: Estimates and projections. PLoS ONE.

[10] Chen S., Bloom D.E. (2019). The macroeconomic burden of noncommunicable diseases associated with air pollution in China. PLoS ONE.

[11] Institute for Health Metrics and Evaluation GBD Compare. https://www.healthdata.org/data-tools-practices/interactive-visuals/gbd-compare.

[12] University of Notre Dame Notre Dame Global Adaptation Initiative. https://gain.nd.edu/our-work/country-index/.

[13] World Health Organization Global Health Risks: Mortality and Burden of Disease Attributable to Selected Major Risks. https://www.who.int/publications/i/item/9789241563871.

[14] GBD 2019 Diseases and Injuries Collaborators (2020). Global burden of 369 diseases and injuries in 204 countries and territories, 1990–2019: A systematic analysis for the Global Burden of Disease Study 2019. Lancet.

[15] Instituto Nacional de Salud Pública Carga de la Enfermedad en México, 1990–2010: Nuevos Resultados y Desafíos. https://www.insp.mx/produccion-editorial/publicaciones-anteriores-2010/3551-carga-enfermedad-mexico.html.

[16] Global Burden of Disease Study 2021 (GBD 2021)—Nonfatal Health Outcomes 2. Analysis for the Estimation of Cause-Specific YLDs by Location, Age, Sex, and Year for GBD 2021—Atrial Fibrillation & Flutter. Global Health Data Exchange. https://ghdx.healthdata.org/gbd-2021/code/nonfatal-2.

[17] Schraufnagel D.E., Balmes J.R., Cowl C.T., De Matteis S., Jung S.H., Mortimer K., Perez-Padilla R., Rice M.B., Riojas-Rodriguez H., Sood A. (2019). Air pollution and noncommunicable diseases: A review by the Forum of International Respiratory Societies’ Environmental Committee, part 2: Air pollution and organ systems. Chest.

[18] Wang Y.L., Lin Y.C., Liu W.C., Lee Y.H., Chiu H.W. (2025). Air pollution and its impacts on health: Focus on microplastics and nanoplastics. Ecotoxicol. Environ. Saf..

[19] Xin X., Chen B., Yang M., Gao S., Wang H., Gu W., Li X., Zhang B. (2023). A critical review on the interaction of polymer particles and co-existing contaminants: Adsorption mechanism, exposure factors, effects on plankton species. J. Hazard. Mater..

[20] Shukla A., Bunkar N., Kumar R., Bhargava A., Tiwari R., Chaudhury K., Goryacheva I.Y., Mishra P.K. (2019). Air pollution associated epigenetic modifications: Transgenerational inheritance and underlying molecular mechanisms. Sci. Total Environ..

[21] Niedzwiecki M.M., Miller G.W., Jirtle R.L. (2022). Molecular mechanisms of environmental exposures and human disease. Nat. Rev. Genet..

[22] Alhuneafat L., Al Ta’aNi O., Arriola-Montenegro J., Al-Ajloun Y.A., Naser A., Chaponan-Lavalle A., Ordaya-Gonzales K., Pertuz G.D.R., Maaita A., Jabri A. (2025). The burden of cardiovascular disease in Latin America and the Caribbean, 1990–2019: An analysis of the global burden of disease study. Int. J. Cardiol..

[23] Macchi C., Sirtori C.R., Corsini A., Mannuccio Mannucci P., Ruscica M. (2023). Pollution from fine particulate matter and atherosclerosis: A narrative review. Environ. Int..

[24] Brauer M., Casadei B., Harrington R.A., Kovacs R., Sliwa K. (2021). Taking a stand against air pollution—The impact on cardiovascular disease. J. Am. Coll. Cardiol..

[25] Vincent R., Kumarathasan P., Goegan P., Bjarnason S.G., Guénette J., Karthikeyan S., Thomson E.M., Adamson I.Y., Watkinson W.P., Battistini B. (2022). Acute cardiovascular effects of inhaled ambient particulate matter: Chemical composition-related oxidative stress, endothelin-1, blood pressure, and ST-segment changes in Wistar rats. Chemosphere.

[26] Ilic I., Ilic M. (2024). The burden of type 2 diabetes mellitus in Latin America, 1990–2019: Findings from the Global Burden of Disease Study. Public Health.

[27] Wang H., Ye X., Zhang Y., Ling S. (2022). Global, regional, and national burden of chronic obstructive pulmonary disease from 1990 to 2019. Front. Physiol..

[28] Reyes-Caballero H., Rao X., Sun Q., Warmoes M.O., Lin P., Sussan T.E., Park B., Fan T.W., Maiseyeu A., Rajagopalan S. (2019). Air pollution-derived particulate matter dysregulates hepatic Krebs cycle, glucose and lipid metabolism in mice. Sci. Rep..

[29] Taylor-Blair H.C., Siu A.C.W., Haysom-McDowell A., Kokkinis S., Saeid A.B., Chellappan D.K., Oliver B.G., Paudel K.R., De Rubis G., Dua K. (2024). The impact of airborne particulate matter-based pollution on the cellular and molecular mechanisms in chronic obstructive pulmonary disease (COPD). Sci. Total Environ..

[30] Quezada-Maldonado E.M., Sánchez-Pérez Y., Chirino Y.I., García-Cuellar C.M. (2021). Airborne particulate matter induces oxidative damage, DNA adduct formation and alterations in DNA repair pathways. Environ. Pollut..

[31] Mendoza-Cano O., Murillo-Zamora E. (2024). Assessing the asthma-related burden of disease in Latin American and Caribbean countries: A sociodemographic perspective. Public Health.

[32] Lu C., Wang F., Liu Q., Deng M., Yang X., Ma P. (2023). Effect of NO_2_ exposure on airway inflammation and oxidative stress in asthmatic mice. J. Hazard. Mater..

[33] Coker E.S., Cavalli L., Fabrizi E., Guastella G., Lippo E., Parisi M.L., Pontarollo N., Rizzati M. (2020). The effects of air pollution on COVID-19 related mortality in Northern Italy. Environ. Res..

[34] Yin P., Gao Y., Chen R., Liu W., He C., Hao J., Zhou M., Kan H. (2023). Temperature-related death burden of various neurodegenerative diseases under climate warming: A nationwide modelling study. Nat. Commun..

[35] World Health Organization (2017). Climate-Resilient Health Systems: A Framework for Action.

[36] Koengkan M., Fuinhas J.A., Silva N. (2021). Exploring the capacity of renewable energy consumption to reduce outdoor air pollution death rate in Latin America and the Caribbean region. Environ. Sci. Pollut. Res..

[37] Dockery D.W., Rich D.Q., Goodman P.G., Clancy L., Ohman-Strickland P., George P., Kotlov T. (2013). HEI Health Review Committee. Effect of Air Pollution Control on Mortality and Hospital Admissions in Ireland.

[38] Tanaka S. (2015). Environmental regulations on air pollution in China and their impact on infant mortality. J. Health Econ..

[39] White P., Conway R., Byrne D., O’Riordan D., Silke B. (2020). Air pollution and comorbidity burden influencing acute hospital mortality outcomes in a large academic teaching hospital in Dublin, Ireland: A semi-ecologic analysis. Public Health.

[40] Yap P.S., Garcia C. (2015). Effectiveness of residential wood-burning regulation on decreasing particulate matter levels and hospitalizations in the San Joaquin Valley Air Basin. Am. J. Public Health.

[41] Yorifuji T., Kashima S., Doi H. (2016). Fine-particulate air pollution from diesel emission control and mortality rates in Tokyo: A quasi-experimental study. Epidemiology.

[42] Viard V.B., Fu S. (2015). The effect of Beijing’s driving restrictions on pollution and economic activity. J. Public Econ..

[43] Zigler C.M., Kim C., Choirat C., Hansen J.B., Wang Y., Hund L., Samet J., King G., Dominici F., HEI Health Review Committee (2016). Causal Inference Methods for Estimating Long-Term Health Effects of Air Quality Regulations.

[44] Rizan C., Bhutta M.F., Reed M., Lillywhite R. (2017). The Impact of surgery on global climate: A carbon footprinting study of operating theatres in three health systems. Lancet Planet. Health.

[45] Eckelman M.J., Sherman J. (2016). Environmental impacts of the U.S. health care system and effects on public health. PLoS ONE.

